# The Pharmacokinetics of Δ^9^-Tetrahydrocannabinol in Sheep

**DOI:** 10.3390/ani14223328

**Published:** 2024-11-19

**Authors:** Sarah A. Stevens, Scott H. Edwards, Glenys K. Noble, Colin J. Scrivener, Gaye L. Krebs, Christopher E. Petzel, Christopher D. May, Zi Xuan Tai, Bronwyn L. Blake, Kenneth C. Dods, Leon N. Warne

**Affiliations:** 1 School of Agricultural, Environmental and Veterinary Sciences, Charles Sturt University, Wagga Wagga, NSW 2650, Australia; sedwards@csu.edu.au (S.H.E.); gnoble@csu.edu.au (G.K.N.); col.scrivener@hotmail.com (C.J.S.); gkrebs@csu.edu.au (G.L.K.); cpetzel@csu.edu.au (C.E.P.); 2 ChemCentre, Bentley, WA 6983, Australia; cmay@chemcentre.wa.gov.au (C.D.M.); atai@chemcentre.wa.gov.au (Z.X.T.); 3 Hemp Feed Solutions, Vasse Valley, North Jindong, WA 6280, Australia; bronwyn@hempfeedsolutions.com.au; 4 SAGE Consultancy, Dianella, WA 6059, Australia; ken.dods@sageconsultancy.com.au; 5 The Vet Pharmacist, East Fremantle, WA 6158, Australia; leon.warne@anaesthesia.vet; 6 Curtin Medical School, Curtin Health Innovation Research Institute, Curtin University, Bentley, WA 6002, Australia; 7 College of Science, Health, Engineering and Education, Murdoch University, Murdoch, WA 6150, Australia

**Keywords:** industrial hemp, THC, pharmacokinetics, residues, ruminants

## Abstract

The pharmacokinetics of Δ^9^-tetrahydrocannabinol (Δ^9^-THC) has not been established in ruminants. Pharmacokinetic knowledge is important given feeding industrial hemp biomass has been shown to result in tissue residues post feeding in sheep. Due to a lack of testing and available data, a ‘maximum’ concentration of Δ^9^-THC has not been currently set for foods of animal origin. Consequently, this study was designed to gain a better understanding of how ruminants process Δ^9^-THC. Eight Merino ewes were administered with two per os (PO) doses of 88.5 mg Δ^9^-THC/kg bodyweight (BW) 12 h apart. Blood samples were collected periodically post dosing to determine the pharmacokinetics of Δ^9^-THC and subcutaneous fat biopsies were taken to investigate the deposition and elimination of Δ^9^-THC from sheep. An elimination half-life of 31.40 ± 13.87 h was identified, with residues persisting in the subcutaneous fat for 28 d in five of the eight sheep, before decreasing below the limit of detection in all sheep by 91 d. These results support the prolonged presence of Δ^9^-THC residues previously identified. Thus, imposing a practical withholding period for ruminants involved in the food chain may not be possible, with further research required to investigate how iHemp biomass may be safely fed to ruminants.

## Simple Summary

Industrial hemp biomass, the low Δ^9^-tetrahydrocannabinol variety of *Cannabis sativa*, has been identified as a potential feed for ruminants. To be a viable option for ruminants involved in the food chain, industrial hemp biomass needs to be suitable for both the animal and consumer. Studies have found industrial hemp biomass to be a nutritionally suitable feed for sheep; however, Δ^9^-tetrahydrocannabinol residues were identified in various tissues post feeding, persisting for up to 140 d in some sheep. Currently, there is zero tolerance for Δ^9^-tetrahydrocannabinol to be present in foods of animal origin (meat, milk, eggs), as no ‘maximum’ level has been set by Food Standards Australia and New Zealand, due to a lack of testing and available data. Consequently, the aim of this study was to investigate how ruminants process Δ^9^-tetrahydrocannabinol and elucidate why Δ^9^-tetrahydrocannabinol persists in animal tissues for an extended period of time. Results from this study support the prolonged residues previously identified in sheep, having implications for the potential utilisation of industrial hemp biomass as a feed for ruminants involved in the human food chain.

## 1. Introduction

Industrial hemp (iHemp) biomass, the low Δ^9^-tetrahydrocannabinol (Δ^9^-THC) variety of *Cannabis sativa* L., was identified as a potential feed option for ruminant animals in Australia (Agrifutures, 2020) due to its quick growth rate and high biomass yield. The current literature regarding feeding of iHemp to livestock focuses predominately upon feeding hemp seed [1,2,3,4] or hemp seed byproducts [5,6,7,8,9,10] which possess very low concentrations of the psychoactive constituent, Δ^9^-THC. In most countries, iHemp biomass cannot be fed to livestock due to the risk of cannabinoid residues accumulating in animal tissues and entering the human food chain [11]. Previously, it was identified that iHemp biomass is a nutritionally suitable feed for sheep following inclusion of iHemp stubble in a pelleted diet up to 56% [12] and iHemp hay in a pelleted diet at 42% [13]; however, cannabinoid residues were present in various tissues post feeding, persisting up to 140 d in some sheep.

Developing guidelines including potential withholding periods for iHemp biomass requires knowledge of how the major cannabinoids present in the biomass are absorbed, metabolised, distributed and eliminated, i.e., the pharmacokinetics, from the ruminant animal. Studies have been conducted analysing the pharmacokinetics and/or effects of Δ^9^-THC in mice [14], rats [15], rabbits [16], pigs [17], parrots [18], dogs [19], cats [20], monkeys [21,22] and humans [23]. Studies investigating cannabinoids in ruminants are limited to those conducted by Jakubovič et al. [24], Cotterill et al. [25], Kleinhenz et al. [26], Krebs et al. [12], Kleinhenz et al. [27], Meyer et al. [28], Stevens et al. [13], Wagner et al. [29], Cornette [30] and Ran et al. [31]. In these studies cattle were either dosed per os (PO) with cannabidiolic acid (CBDA) [26] or cannabidiol (CBD) [28,30], or fed diets containing either iHemp biomass [27] or iHemp silage [29]; while sheep were intravenously (IV) dosed with up to 1 mg Δ^9^-THC/kg bodyweight (BW) [24,25] or fed diets containing either iHemp stubble [12] or iHemp hay [13]; and goats were fed diets containing iHemp biomass [31]. These ruminant studies presented neither comprehensive pharmacokinetic data of Δ^9^-THC nor determined the bioavailability or safety of Δ^9^-THC (when delivered in relatively high doses as could occur under some grazing conditions) in ruminants.

Pharmacokinetic knowledge is particularly important when a feed results in tissue residues post feeding, as residues may restrict product incorporation into the human food chain. Currently, a ‘maximum’ level of Δ^9^-THC has been set by Food Standards Australia and New Zealand (FSANZ) only for foods of plant origin [32]. Due to a lack of testing and available data, FSANZ has not set the maximum level for Δ^9^-THC in foods of animal origin [33]. Some of the required data are the pharmacokinetics of Δ^9^-THC. Due to species variation in the absorption, distribution, metabolism and elimination of Δ^9^-THC, the pharmacokinetics of Δ^9^-THC must be elucidated in ruminants for progress to be made in the development of guidelines, such as withholding periods, to allow feeding of iHemp biomass to livestock involved in the food chain. Determining the pharmacokinetics is just the first step, with residue depletion studies in all edible tissues also required.

Therefore, the aim of this study was to describe the pharmacokinetics of Δ^9^-THC using a Δ^9^-THC containing resin administered PO to sheep and to investigate the deposition and elimination of Δ^9^-THC from subcutaneous fat tissue to generate data that will aid in the development of guidelines for the use of iHemp biomass as a feed for ruminants, especially if they are destined for the food chain.

## 2. Materials and Methods

The study was conducted at the Department of Primary Industry (DPI) Animal Nutrition Unit at Wagga Wagga, New South Wales (NSW), Australia. The use and care of animals for the project was approved by Charles Sturt University’s (CSU) Animal Ethics Committee (Protocol number: A21096 and A22440) and by the DPI Animal Care and Ethics Committee (Protocol number: ORA 21/24/014C and OAEC-0547). The study was compliant with the Animal Research Act 1985 (as amended) in accordance with the Australian Code of Practice for the Care and Use of Animals for Scientific Purposes.

### 2.1. Pilot Study

**Figure 1:**
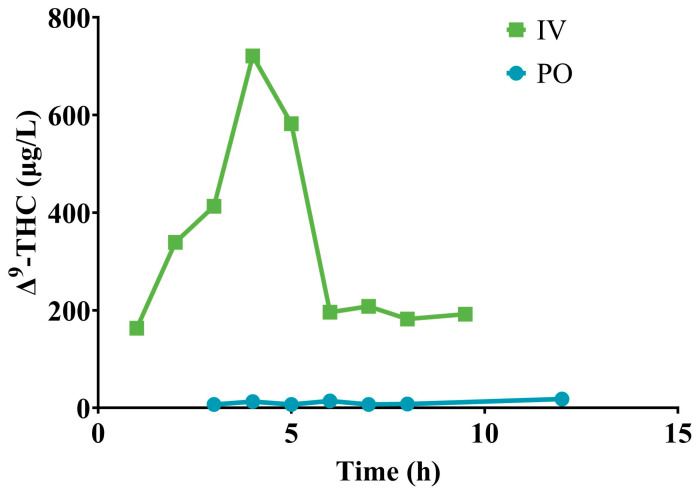
Plasma concentrations of Δ^9^-THC at various times after either a single IV or single PO dose of 18.7 mg Δ^9^-THC/kg BW to two Merino ewes.

Two doses, one delivered by IV and one delivered by PO, were investigated in a pilot study to establish the bioavailability of Δ^9^-THC in sheep. The PO dose was provided as a single PO bolus whilst the IV dose was titrated over 5.5 h. Two Merino ewes (48 kg and 49 kg, liveweight) of approximately two years of age were randomly assigned to receive either an IV dose of 18.7 mg Δ^9^-THC/kg BW or a PO dose of 18.7 mg Δ^9^-THC/kg BW. Blood samples were collected from a catheter inserted in the jugular vein for the analysis of plasma for Δ^9^-THC at 0 h, 1 h, 2 h, 3 h, 4 h, 5 h, 6 h, 7 h and 8 h post-dose administration in both sheep, and a further sample collected at 9.5 h for the IV sheep and at 12 h for the PO sheep. Based on the area under the curve (AUC) for both the IV and PO dose, the bioavailability of Δ^9^-THC in sheep was approximately 5% (Figure 1). It must be emphasised that this is an approximate value based on early data, as to confirm bioavailability plasma concentrations of Δ^9^-THC would have needed to be tracked for a longer duration of time; however, due to the death of the sheep administered IV with Δ^9^-THC at the 9.5 h timepoint, further sampling was not possible.

### 2.2. Oral Dose Study

From the pilot study, it was determined that to calculate the pharmacokinetics of Δ^9^-THC in sheep, a dose greater than 18.7 mg Δ^9^-THC/kg BW would be required, and it would have to be administered by PO due to safety concerns following IV administration. To calculate an appropriate dosage, the worst-case grazing scenario was considered where all tetrahydrocannabinolic acid (THCA) in the plant is converted into Δ^9^-THC. The dosage was calculated based on the average amount of Δ^9^-THC that would be consumed by a sheep grazing an iHemp crop at the maximum allowable limit, being 1% in NSW (Hemp Industry Act 2008). Previously, adult Merino ewes fed a pelleted ration containing 42% iHemp hay consumed 3.6% of their BW on a dry matter (DM) basis [13]. Based off the bioavailability calculated in the pilot study (and to be on the safe side), a 50% reduction in dosage was implemented, and was based on sheep consuming 3.5% of their BW, for a final dosage of 175 mg Δ^9^-THC/kg BW (upon analysis of the dose solution, the actual average administered dose was 177 mg Δ^9^-THC/kg BW). If animals were grazing iHemp or fed an iHemp-containing ration, they would consume the total dose of ∆^9^-THC over a 24 h period. To better align to feeding and also as a precautionary measure, the ∆^9^-THC was delivered as two PO boluses (half of total dose per bolus) administered 12 h apart.

#### 2.2.1. Experimental Animals, Housing and Diets

Eight Merino ewes (47.8 ± 3.49 kg; mean ± SD), of approximately 24 months of age were sourced from the same flock, selected based upon age, body condition score and BW, to minimise any potential differences in the composition of their (initial) rumen microbial populations and to minimise any differences that might occur due to environmental adaptation. All sheep were determined to be clinically healthy based on a physical examination conducted by a registered veterinarian prior to their recruitment in the project. The sheep were housed indoors, in individual pens (1.2 × 2.4 m) for the duration of the experiment. The sheep were released out into a nearby paddock 7 d after the 28 d subcutaneous fat biopsy was taken and remained there until subsequent subcutaneous fat biopsies returned negative for cannabinoid residues for all sheep. For the entire experimental period, the sheep had access to fresh, clean water. The sheep were closely monitored throughout the study period and vital parameters were assessed when abnormal behaviour or clinical signs were observed.

The sheep were fed a maintenance plus ration consisting of wheaten chaff (Corowa Chaff Mills, Corowa, NSW, Australia) and a commercial sheep pellet (Ewe and Lamb Nuts, Laucke Mills, South Australia, Australia) once daily at 0900. The sheep were adapted onto the diet as follows: Day 1: 20% pellets + 80% chaff; Day 3: 40% pellets + 60% chaff; Day 6 onwards: 50% pellets + 50% chaff. The daily ration was provided ad libitum (known quantity).

#### 2.2.2. Experimental Design

The experiment consisted of one experimental treatment with eight replicates [26,34]. The treatment was two PO doses of 88.5 mg Δ^9^-THC/kg BW administered 12 h apart. Sheep were randomly allocated to an individual pen and a pilot animal was randomly selected. A pilot study involving one sheep was undertaken to ensure the dose was safe as well as to define the appropriate time points for blood and subcutaneous fat sample collection for the remaining sheep involved in the study. The experimental period comprised a total of 62 d including a dietary and housing adaptation of 12 d for the pilot study and 34 d for the remaining 7 sheep (time it took for the results to be available from the pilot study), followed by a 28 d sample collection period.

On the day of and prior to PO administration of the cannabinoid solution, each sheep was instrumented with a 16-gauge IV catheter to facilitate serial blood sampling. All sheep were dosed according to BW. The required amount of a 73.2% Δ^9^-THC resin (Little Green Pharma^®^, Western Australia, Australia) was weighed using calibrated scales (Sartorius, Germany), then dissolved in dimethyl sulfoxide (DMSO). All doses were administrated via an orogastric tube, followed by 10 mL of DMSO to ‘flush’ the tube to ensure full delivery of the dose solution. A new orogastric tube was used for each sheep to ensure there was no cross contamination.

#### 2.2.3. Sampling Protocol

Blood samples for plasma cannabinoid analysis were collected via the 16-gauge catheter located in the right jugular vein. Prior to administration of the dose solution, a blood sample (0 h) was collected from each animal. Following the PO bolus, blood sampling occurred at 12 h, 24 h, 36 h, 48 h, 72 h, 96 h, 120 h, 144 h, 168 h, 192 h, 216 h, 240 h, 264 h, 288 h, 312 h, 336 h, 504 h and 672 h. For each sample, the initial 3 mL of blood collected was discarded and a sterile syringe was used to draw 10 mL of blood that was then placed into a vacutainer containing lithium heparin and immediately inverted. The catheter was maintained with approximately 10 mL of a 10,000 IU heparin saline solution following the collection of each sample. Within 30 min of collection, the samples were centrifuged at 1300 rcf for 10 min at room temperature (approximately 24 °C). The plasma was then harvested into 5 mL screw-top tubes and stored frozen at −18 °C for subsequent cannabinoids analyses.

Incisional subcutaneous fat biopsies were conducted by a registered veterinarian, as per Stevens et al. [13], at 14 d, 28 d and 56 d post dosing for the pilot sheep and at 28 d and 91 d post dosing for the remaining 7 sheep. Each time, the sample was divided into two, with each subsample placed in a 50 mL screw-top specimen container and stored at −18 °C until cannabinoid analysis could occur. Following each fat biopsy, the sheep were monitored closely for the next 7 d to ensure the site of incision did not become infected and healed appropriately.

#### 2.2.4. Cannabinoids Analysis

Analysis for cannabinoid concentrations in both the plasma and subcutaneous fat was conducted by ChemCentre Western Australia. All subcutaneous fat and plasma samples were stored at −18 ± 2 °C before analysis.

##### Sample Preparation

Subcutaneous fat. Subcutaneous fat samples were freeze-dried before being cut into small pieces (ca. 2 mm) using a scalpel. Approximately 0.1 g of each sample was suspended in 10 mL of ice-cold methanol (−18 ± 2 °C) in a 15 mL centrifuge tube. The samples were immediately vortexed and then sonicated (<10 °C cold water bath) for 10 min to aid extraction. The samples were placed into a centrifuge at 2200 rcf for 10 min at room temperature before a 2 mL aliquot of the supernatant was transferred to a glass test tube. Deuterated internal standards (CBD-d3, Δ^9^-THC-d3, 11-nor-9-carboxy-Δ^9^-THC(THC-COOH)-d3) and quality control spikes produced by an ISO 17034 [35] supplier (Cerilliant Corporation, Round Rock, TX, USA) were added to the corresponding tubes. All tubes were then gently mixed using a vortex mixer. The mixture was passed through an SPE column (Oasis Prime HLB Plus Light Cartridge (Waters Corporation, Milford, MA, USA)) for clean-up prior to LC-MS/MS analysis.

Plasma. Approximately 100 µL of the plasma sample was pipetted and extracted with 5 mL of iced-cold methanol in a 10 mL centrifuge tube. Samples were immediately vortexed to mix for 2 min to assist with dissolution. Following centrifugation at 2200 rcf for 10 min at room temperature, a 2 mL aliquot of the supernatant was pipetted and transferred into a labelled mini centrifuge tube. Deuterated internal standards (CBD-d3, Δ^9^-THC-d3, THC-COOH-d3) and quality control spikes (Cerilliant Corporation) were added to the corresponding tubes and mixed using a vortex mixer, followed by centrifugation at 7000 rcf for 10 min at room temperature. The Oasis Prime HLB Plus Light Cartridge was then used for pass-through cleanup of the mixture in the manual mode with a syringe on a dropwise flow, and a portion of the cleaned extract was vialed up for analysis using LC-MS/MS.

##### LC-MS/MS Conditions

The analysis was performed on an Agilent 1290 Infinity II LC (Santa Clara, CA, USA) coupled to an Agilent 6470 MS/MS.

LC Conditions. The LC column was Agilent Poroshell 120 EC-C18 1.9 µm 2.1 × 100 mm with Agilent Poroshell 120 EC-C18 2.1 mm guard column. The injection volume was 3 µL. The mobile phase A = 0.1% formic acid in water and B = 0.1% formic acid in methanol, at a flow rate of 0.4 mL/min. The LC run time was 17.5 min and the column heater temperature was 50 °C.

**Figure 2:**
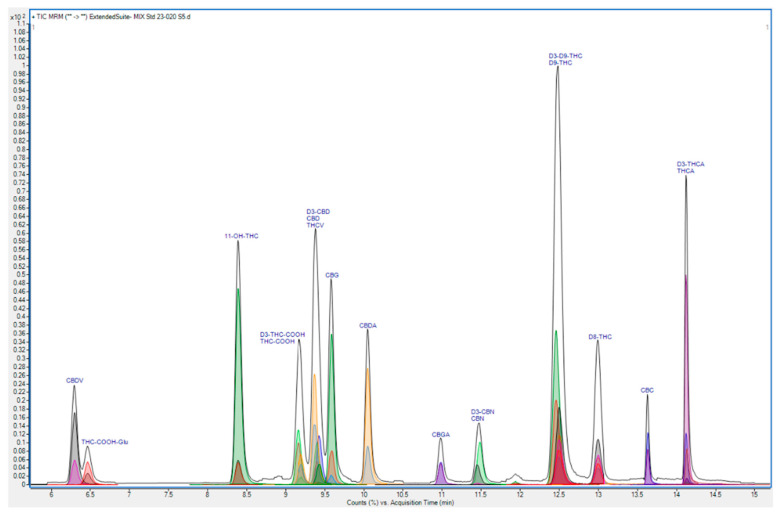
Total ion chromatogram comprising MRM transitions for cannabinoid and metabolite analytes in in-house analytical method ORG179.

MS Conditions. The desolvation gas was UHP nitrogen. The polarity was positive ion mode. The mass scanning mode was dynamic multiple reaction monitoring (dMRM). The gas temperature was 300 °C and the flow was 10 L/min. The nebulizer was 35 psi, the sheath gas temperature was 300 °C and the sheath gas flow was 10 L/min. These conditions were the outcome of in-house method optimisation using instrument manufacturer software. A total ion chromatogram comprising the MRM transitions for the analytes of interest is included in Figure 2. The quantifier and qualifier MRM transitions for the analytes of interest are listed in Table 1.

All analytical results were calibrated against certified reference materials produced by an ISO 17034 supplier (Cerilliant Corporation).

##### Method Validation—Plasma

The in-house analytical method ‘ORG179 Determination of Cannabinoids and Metabolites by LC-MS/MS’ was validated based on the guidelines provided by Eurachem 2nd ed. [36] and NATA Technical Note 17 [37]. Comprehensive performance characteristics were considered in the process including specificity, linearity, limit of detection (LOD), limit of quantification (LOQ), accuracy and precision.

The specificity of the method was determined by analysing the blank plasma matrix and a calibration standard was run. Linearity range was studied based on a calibration plot ranging from 0 to 100 µg/L for both phytocannabinoids and the THC metabolites. The method detection limit was obtained by analysis of at least seven replicates of the lowest concentration (0.5 µg/L) calibration standard and method blank. A matrix blank was also performed to compare baseline readings and matrix effects. Method accuracy and precision were demonstrated by a minimum of seven replicate analyses of high and low spikes of known concentrations into the blood sample, and the results were pooled.

Compound identification was based upon comparison to certified standards (Cerilliant Corporation) matching a minimum of two mass spectrometry transitions, qualifier ratios and retention time data. No cannabinoids or metabolites were detected in the blood matrix blank and reagent blank and thus indicated the specificity of the procedure for the targeted analytes. The method working range was subsequently refined to a range from 0 to 20 µg/L for phytocannabinoids and the THC metabolites. The generated calibration curves were fit for purpose with a minimum correlation coefficient (R^2^) of 0.995 across all analytes applying quadratic regression across seven data points with no extrapolation of results beyond the top calibration standard. LOD and LOQ were calculated based upon 3 and 10 standard deviations of the blank, respectively. The limit of reporting (LOR) of the method was set at 5 µg/L for the cannabinoids and metabolites after a dilution factor was considered (Table 2).

Method accuracy and precision were determined at two concentrations of each analyte with a minimum of seven replicates of each concentration. All individual spike recovery of targeted cannabinoids and metabolites varied from 75% to 110%, while relative standard deviation percentages (%RSD) remained less than 10%. The summarised accuracy and precision data are provided in Table 3.

#### 2.2.5. Pharmacokinetic Analysis

Maximum measured concentration (Cmax) and time to maximum concentration (Tmax) of Δ^9^-THC were determined directly from the data. Other pharmacokinetic parameters from Δ^9^-THC concentration vs. time data were determined for each sheep by use of noncompartmental analysis with a commercial software program (PKSolver).

The AUC_0–∞_ and area under the first moment curve (AUMC_0–∞_) were calculated by use of the linear trapezoidal rule. Mean residence time (MRT_0–∞_) was calculated as AUMC_0–∞_/AUC_0–∞_, where AUMC_0–∞_ is the area under the first moment curve from 0 to infinity. Terminal elimination half-life was calculated as (ln 2)/λz, where λz is the terminal elimination rate constant calculated by means of log-linear regression. Calculation of the terminal elimination half-life involved the use of the last four to six data points for each sheep. Apparent oral clearance and volume of distribution were calculated as Cl/F and Vd/F, respectively, where F is the approximate oral bioavailability of Δ^9^-THC in sheep.

## 3. Results

### 3.1. Pharmacokinetic Calculations

**Figure 3:**
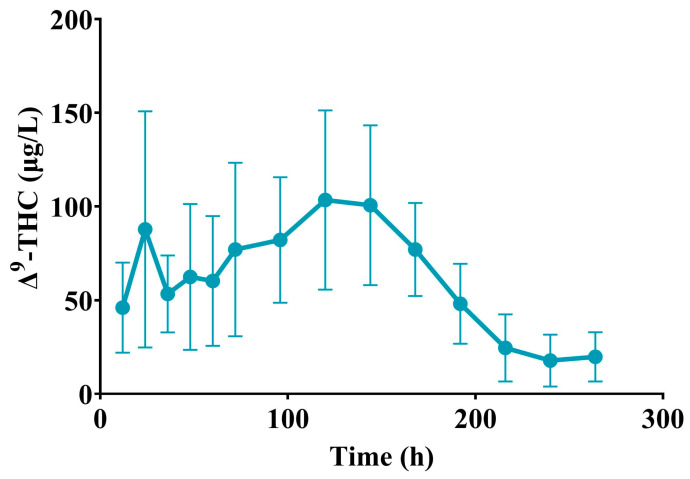
Mean ± SD plasma concentration of Δ^9^-THC at various times after PO administration to 8 adult Merino ewes at a dose of 177 mg/kg BW.

For all eight sheep, Δ^9^-THC was detectable in the plasma at 216 h, but by 264 h, Δ^9^-THC was only detectable in the plasma of three sheep. No metabolites of Δ^9^-THC were detectable in the plasma of any sheep at any timepoint. Mean ± SD elimination half-life of Δ^9^-THC was 31.40 ± 13.87 h, mean apparent clearance was 0.23 ± 0.06 L/h/kg and mean apparent volume of distribution was 10.17 ± 4.39 L/kg. The pharmacokinetic data are summarised in Table 4. The Δ^9^-THC concentration time curve after PO administration was plotted (Figure 3).

### 3.2. Subcutaneous Fat Cannabinoid Concentrations

For the pilot sheep (*n* = 1), Δ^9^-THC (5814 µg/kg) was detectable in the subcutaneous fat 14 d post dosing but was undetectable in the subcutaneous fat 28 d and 56 d post dosing. For the remaining seven sheep, four sheep had a detectable level of Δ^9^-THC (21.63, 67.28, 81.08 and 227.57 µg/kg) in the subcutaneous fat 28 d post dosing, but by 91 d post dosing, no sheep had a detectable level of Δ^9^-THC present in the subcutaneous fat.

## 4. Discussion

### 4.1. Pharmacokinetics

Maximum Δ^9^-THC plasma concentrations generally occur 2 h post PO dosing in humans; however, they may be observed 4 h to 6 h later [23]. A similar timeframe of 2.5 h was identified in parrots [18]. In the current study, the mean ± SD maximum concentration of Δ^9^-THC occurred 97.50 ± 65.55 h post administration of the first half of the dose. The increased Tmax identified in the current study may be attributed to the split dose, administered 12 h apart. The increased Tmax aligns with that identified in humans following multiple PO doses of Δ^9^-THC, with an increase from 3 h after a single dose to 103.5 h following multiple doses [38]. Additionally, the increased Tmax was indicative of Δ^9^-THC most likely being absorbed from the small intestine rather than the rumen, as it took an average of 4 d to reach maximum concentrations in the plasma. In terms of a grazing situation, the results from this study indicated that the concentration of Δ^9^-THC in the plasma may slowly increase over the grazing period and result in potentially greater plasma concentrations than those identified in the current study.

As the dose in the current study was administered by PO, apparent volume of distribution and clearance represent theoretical values only and were based off a bioavailability of approximately 5% for Δ^9^-THC in sheep. As the bioavailability was calculated from a pilot study involving only two sheep, one sheep dosed IV with Δ^9^-THC and the other dosed PO with Δ^9^-THC, it must be emphasised that both the apparent volume of distribution and clearance calculated in the current study are approximate values only. The mean ± SD volume of distribution found in the current study was 10.17 ± 4.39 L/kg. In humans, the volume of distribution of Δ^9^-THC has been reported, on average, as 5 L/kg [39] up to 10 L/kg [40], similar to the value calculated in the current study. Conversely, in pigs, a much greater mean ± SD volume of distribution of 32.0 ± 14.6 L/kg was identified [17], possibly linked to differences in fat composition between pigs and sheep [41]. The mean ± SD clearance found in the current study was 0.23 ± 0.06 L/h/kg. Comparatively, the clearance rate was 45.6 to 71.6 L/h (or 0.65 to 1.0 L/h/kg assuming an average BW of 70 kg) in humans [42], 1.56 ± 0.6 L/h/kg in rabbits [16] and 2.2 ± 0.6 L/h/kg in pigs [17]. The lower clearance rate of 0.23 ± 0.06 L/h/kg identified in the current study may be attributed to species differences in the plasma protein binding of Δ^9^-THC.

A reasonably high dose of Δ^9^-THC was utilised in the current study to ensure terminal elimination half-life could be characterised from the concentration time curve. The mean ± SD terminal elimination half-life of Δ^9^-THC in sheep was 39.5 ± 23.9 h, which was within the same range as the mean half-life of 25 to 36 h reported in humans [40], and 47.1 ± 3.5 (SE) (range 30–66) h reported in rabbits [16]; however, it was longer than the half-life of 10.6 ± 2.2 h reported in pigs [17] and 4.58 ± 8.43 h reported in parrots [18]. The rate-limiting step for the elimination of unchanged Δ^9^-THC has been suggested to be due to the slow return of previously tissue sequestered Δ^9^-THC to plasma rather than due to metabolism [43]. The long terminal elimination half-life identified in the current study aligned with the long period of detection of Δ^9^-THC residues, up to 140 d, when sheep were fed a diet containing iHemp hay which delivered an average daily dose of 4.7 mg total THC/kg BW [13]. The long elimination half-life of Δ^9^-THC has implications in terms of the possibility of setting a practical withholding period for animals entering the human food chain. Consequently, other ways to mitigate the risk of residues, such as feeding to particular classes of animals, will have to be investigated if iHemp biomass is to be utilised as a future feed option for ruminants involved in the human food chain under current FSANZ guidelines.

### 4.2. Subcutaneous Fat Cannabinoid Concentrations

Following absorption, Δ^9^-THC rapidly distributes to body tissues, especially those that are highly perfused such as the lung, heart, brain and liver, before preferentially storing in the fat depots of the body due to Δ^9^-THC being a highly lipophilic molecule [44]. In the current study, 50% of the sheep PO dosed with Δ^9^-THC had a detectable level of Δ^9^-THC in the subcutaneous fat 28 d post dosing; however, by 91 d, all eight sheep had undetectable levels of Δ^9^-THC. Δ^9^-THC was detectable in both the kidney and subcutaneous fat of all sheep at slaughter fed a diet containing iHemp stubble for 56 d [12]. Further, Δ^9^-THC residues were present in the subcutaneous fat of sheep up to 140 d post feeding with a diet containing iHemp hay for 21 d [13]. The prolonged time of detection of Δ^9^-THC residues in the subcutaneous fat aligned with the long mean ± SD elimination half-life of 39.5 ± 23.9 h identified in the current study. The difference in time of detection between the sheep PO dosed in the current study and those consuming the iHemp hay was likely due to the difference in Δ^9^-THC intake, i.e., acute vs. chronic. Repeated dosing with Δ^9^-THC to humans [45], parrots [18] and rabbits [16] resulted in an increased elimination half-life and prolonged period of cannabinoid excretion in comparison to a single dose [46]. Further research is required to assess possible practical guidelines that could be implemented if iHemp biomass is to be a possible feed for ruminants involved in the human food chain in the future, taking into account the long duration that Δ^9^-THC resides in the body post exposure.

### 4.3. Signifcance of Findings

The results from this study may aid in the future development of withholding periods for ruminants destined for the food chain that have been fed iHemp biomass. Plasma pharmacokinetics alone cannot determine withholding periods, and therefore, a residue depletion study analysing all edible tissues at a range of different intervals is required. The plasma pharmacokinetic data from the current study will aid in the study design of any future residue depletion studies and act as a cross-validation reference for the tissue residue results.

## 5. Conclusions

The mean ± SD elimination half-life of Δ^9^-THC in sheep identified in the current study was 39.5 ± 23.9 h, which supported previous studies where residues persisted for an extended period post-feeding iHemp biomass. Based on the long elimination half-life of Δ^9^-THC and the presence of tissue residues, more research needs to be conducted, including residue depletion studies, before any hemp-derived products should be fed to ruminants involved in the food chain.

## Figures and Tables

**Table 1:** Analytes and respective MRM transitions for cannabinoids and metabolites of interest.

Compound	Abbreviation	Quantifier	Qualifier 1	Qualifier 2
Cannabidiol	CBD	315.2 → 123.2	315.2 → 193.2	
Δ^9^-tetrahydrocannabinol	Δ^9^-THC	315.2 → 123.1	315.2 → 193.2	315.2 → 259.1
11-nor-9-carboxy-Δ^9^-THC	THC-COOH	345.2 → 193.1	345.2 → 299.1	345.2 → 327.1
11-hydroxy-Δ^9^-THC	11-OH-THC	331.2 → 193.1	331.2 → 201.0	331.2 → 313.1

**Table 2:** Limits of detection, quantification and reporting for analytes of interest.

Analyte	SD (µg/L)	LOD (µg/L)	LOQ (µg/L)	LOR (µg/L)
CBD	0.015	0.05	0.15	5
Δ^9^-THC	0.001	0.003	0.0.01	5
THC-COOH	0.017	0.05	0.17	5
11-OH-THC	0.009	0.03	0.09	5

**Table 3:** Accuracy and precision data for analytes of interest.

Analyte	Spiking Level (µg/L)	Number of Replicates (*n*)	Accuracy	Precision
			% Recovery	% RSD
CBD	10	9	99.3	6.33
	100	7	93.8	8.86
Δ^9^-THC	10	10	88.4	4.52
	100	7	92.2	6.16
THC-COOH	10	10	89.7	6.91
	100	10	89.2	6.42
11-OH-THC	10	10	80.3	6.10
	100	10	77.5	5.86

**Table 4:** Pharmacokinetic parameters of Δ^9^-THC after PO administration to 8 healthy adult Merino ewes at an average dose of 177 mg/kg BW.

Parameter	Mean ± SD	Median (Range)
Cmax (µg/L)	132.88 ± 47.99	126.50 (91.50–222.00)
Tmax (h)	97.50 ± 65.55	132.00 (12.00–168.00)
λz (1/h)	0.03 ± 0.01	0.02 (0.01–0.03)
Terminal elimination half-life (h)	31.40 ± 13.87	28.24 (20.54–60.24)
AUC_0–t_ (µg•h/L)	15,723.36 ± 4116.32	15,606.66 (10,991.34–22,951.02)
AUC_0–∞_ (µg•h/L)	16,525.32 ± 4356.18	15,952.74 (11,226.33–23,122.90)
AUC extrapolated (%)	4.61 ± 4.65	2.58 (0.74–14.97)
MRT_0–∞_ (h)	123.22 ± 22.72	123.43 (95.53–165.14)
Cl/F (L/h/kg)	0.23 ± 0.06	0.22 (0.15–0.32)
Vd/F (L/kg)	10.17 ± 4.39	9.56 (4.58–17.17)

Cmax = maximum concentration. Tmax = time to maximum concentration. λz = terminal elimination rate constant. AUC_0–t_ = area under the curve from 0 to last quantifiable time point. AUC_0–∞_ = area under the curve from 0 to infinity. MRT_0–∞_= mean residence time extrapolated to infinity. Cl = apparent clearance. F = approximate bioavailability of 5%. Vd = apparent volume of distribution.

## Data Availability

The data presented in this study are available on request from the corresponding author.
